# Neuro-immune interactions at single-cell resolution in neurodevelopmental, infectious, and neurodegenerative diseases

**DOI:** 10.1080/19768354.2022.2110937

**Published:** 2022-08-11

**Authors:** Hyun Jung Park, Hosung Jung

**Affiliations:** a Samsung Medical Center, Samsung Genome Institute, Seoul, Republic of Korea; b Department of Anatomy, Graduate School of Medical Science, Brain Korea 21 Project, Yonsei University College of Medicine, Seoul, Republic of Korea

**Keywords:** Single-cell RNA sequencing, COVID-19, multiple sclerosis, Alzheimer's disease, Parkinson's disease

## Abstract

Recent technological advance in single-cell and single-nucleus transcriptomics has made it possible to generate an unprecedentedly detailed landscape of neuro-immune interactions in healthy and diseased brains. In this review, we overview the recent literature that catalogs single-cell-level gene expression in brains with signs of inflammation, focusing on maternal immune activation, viral infection, and auto-immune diseases. The literature also includes a series of papers that provide strong evidence for immunological contributions to neurodegenerative diseases, which, in a strict sense, are not considered neuroinflammatory. To help with the discussion, we present a diagram of experimental and analytical flows in the single-cell analysis of the brain. We also discuss the recurring themes of neuro-immune interactions and suggest future research directions.

## Introduction

The blood–brain barrier prevents peripheral cells and the substances in the extracellular environments of the body from entering the central nervous system (CNS) (Daneman and Prat 2015). However, it has become clear that the peripherally initiated inflammation can spread to the CNS during infection (Manabe and Heneka 2021) and in auto-immune disorders (Karpus 2020; Attfield et al. 2022), leading to the degeneration of neurons and the deterioration of brain function (Huang et al. 2021). Furthermore, brains with neurodegenerative diseases show signs of neuroinflammation, which correlate with the severity of the diseases (Colonna and Brioschi 2020; Haage and De Jager 2022; Tansey et al. 2022).

Cytokines are a family of small, secreted proteins orchestrating the proliferation and migration of immune cells during inflammation (Salvador et al. 2021). Microglia, the resident macrophages of the CNS, express cytokine receptors. Using cytokine signaling, microglia actively participate in synapse remodeling during development (Li Q and Barres 2018), as well as in phagocytosis and cytokine secretion during inflammation in adulthood. The brain cells of neuroepithelial origin, such as neurons (Prieto and Cotman 2017), astrocytes (Choi SS et al. 2014), and oligodendrocytes (Omari et al. 2005), also express cytokine receptors. Hyperactivation of cytokine receptors in neurons and oligodendrocytes usually leads to their degeneration, which is often accelerated by activated astrocytes (Salvador et al. 2021). Because almost all brain cell types can participate in neuroinflammation, a systematic analysis of cell states (*e.g.* expressing ‘homeostatic’ or ‘inflammatory’ genes) at single-cell resolution is required to gain a global understanding of neuro-immune interactions in healthy and diseased brains.

Recent advances in single-cell and single-nucleus RNA sequencing technologies allow exactly this type of investigation to be performed (Choi YH and Kim 2019), and recent studies using mouse and human brains have reported comprehensive datasets with new insights. In this focused review, we summarize recent papers that presented datasets that others can browse and use to formulate new hypotheses. First, we present a general experimental and analytical flow of single-cell and single-nuclei transcriptomic analyses as a framework for discussion to follow.

## Techniques

### Generation of single-cell and single-nucleus sequencing data

Single-cell sequencing (scRNA-seq) is based on the cell-by-cell reverse transcription in droplets, made possible by merging one water droplet containing barcoded oligo-dT primers with the other containing a single cell in a microfluidic flow of oil. cDNAs tagged with cell-specific barcodes are mixed, amplified, and converted to a sequencing library (Choi YH and Kim 2019). The sequencing libraries generated from control (*e.g.* healthy) and experimental (*e.g.* diseased) groups are sequenced together to minimize batch effects.

**Figure 1.**
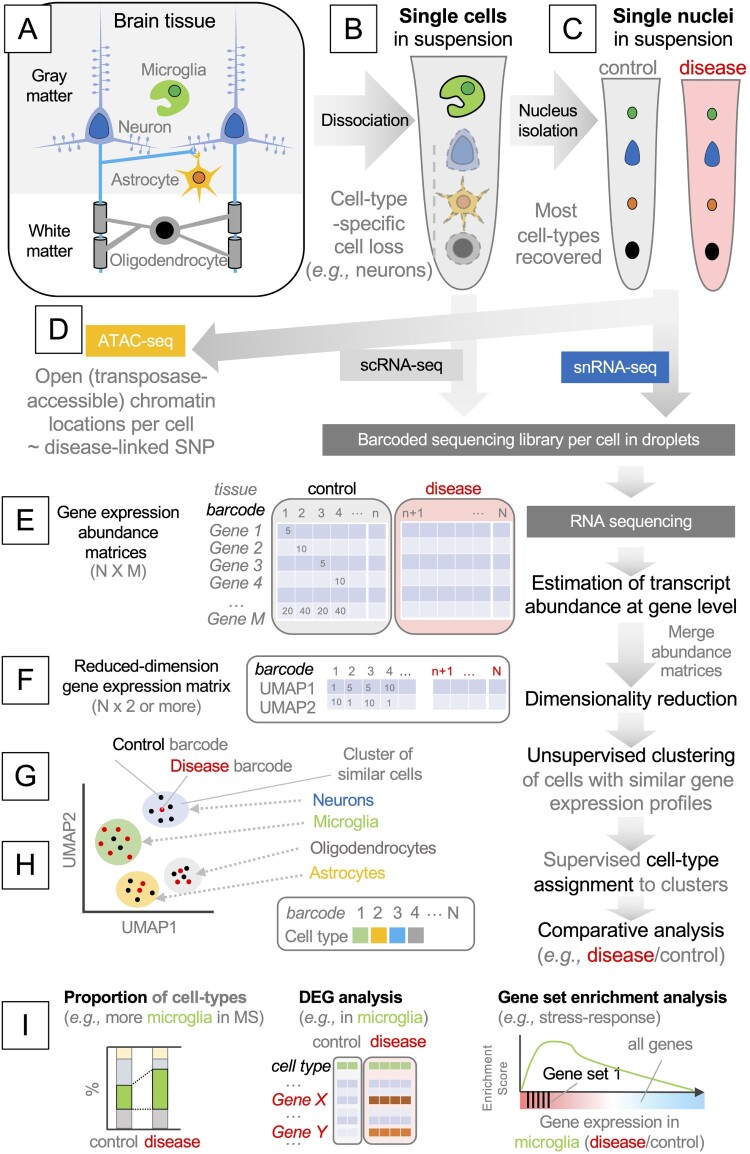
**A highly simplified workflow of single-cell and single-nucleus isolation, RNA sequencing, and bioinformatic analysis.** **(A)** Major cell types in the brain. Generally, gray and white matters contain neuronal cell bodies and axons, respectively. **(B)** Dissociating brain tissue disrupts the plasma membrane of most brain cells of neuroepithelial origin, and microglia become over-represented in a cell suspension. **(C)** The nuclear envelope survives dissociation, and the nuclei of most brain cells are represented in a nucleus suspension. **(D)** Isolated nuclei can be analyzed by assay for transposase-accessible chromatin sequencing (ATAC-seq). **(E)** Single-cell or single-nucleus RNA sequencing produces a gene abundance matrix per sample, where the columns represent cell-specific barcodes, and the rows represent the genes. **(F)** To group cells with a similar gene expression status into a cluster, non-linear dimensionality reduction techniques are used, such as Uniform Manifold Approximation and Projection (UMAP). In this example, gene expression status is now represented by two axes, UMAP1 and UMAP2. **(G)** Neighboring cells are grouped into clusters. **(H)** Each cluster is assigned with a biologically relevant label, such as cell type or status. **(I)** Differences between control and diseased brains are analyzed, usually using gene abundance matrices in (E). DEG: differentially expressed gene.

The first experimental step is to dissociate the brain tissue into single cells. An important premise of single-cell analyses is that the cellular composition of the original tissue remains after dissociation. However, most brain cells, such as neurons, astrocytes, and oligodendrocytes, have extensive cytoplasmic protrusions, which are lost during dissociation (Figure 1A). For example, over 99% of the cytoplasm of a neuron may localize to its axon and dendrites. As such, the cytoplasmic contents of the major cell types of the adult brain are lost during dissociation, and their mRNAs are not recovered in scRNA-seq (Liu et al. 2021) (Figure 1B). This problem was recently resolved by using single nuclei rather than single cells. The nuclear envelope remains intact during dissociation, and the mRNA contents within the nucleus surprisingly well reflect those of the whole cell (Grindberg et al. 2013) (Figure 1C). Therefore, the latest single-cell studies of the adult brain utilized single nucleus RNA-sequencing (snRNA-seq) rather than scRNA-seq. A major exception is microglia. Like their peripheral cousins, microglia retain relatively round morphology with an ample perinuclear cytoplasmic space, and their mRNAs are recovered well in scRNA-seq (Figure 1B). Another exception is the fetal brain (Li Z et al. 2020). As it mainly contains undifferentiated neuronal and glial progenitors with simpler morphologies, the mRNAs of most major brain cell types are recovered in scRNA-seq (Darmanis et al. 2015; Polioudakis et al. 2019; Eze et al. 2021; La Manno et al. 2021).

Optionally, isolated single nuclei can be used in the assay for transposase-accessible chromatin sequencing, or ATAC-seq, which generates sequencing reads of DNA fragments tagged with a cell-specific barcode by experimentally-added transposases (Buenrostro et al. 2015). The assay probes chromosomal regions that have increased accessibility, presumably due to extensive transcriptional regulation (Buenrostro et al. 2013). When combined with the matching snRNA-seq data, this approach identifies putative promoters and enhancers that act in a cell-type-specific manner to increase or decrease the transcription of particular genes (Baek and Lee 2020) (Figure 1D).

### Analysis of single-cell and single-nucleus sequencing data

scRNA-seq and snRNA-seq data are composed of short RNA sequence read pairs, with one read mainly representing the barcode and the other representing the gene body. The first step in the analysis is to generate a gene expression abundance matrix, with a column representing a barcode (*i.e.* the cell from which the read originates) and a row representing a gene (among all annotated genes in the genome). Typically, sequencing reads are aligned to the genome or mapped to the transcriptome to estimate the abundance of each transcript, which is then summarized to the gene level (*i.e.* abundances of transcripts belonging to the same gene are summed) (Figure 1E). The readers are referred to more specialized reviews for the detailed methods (Hwang et al. 2018).

The next step is to determine the cell type of each cell. The cell-typing typically involves the grouping of cells with similar gene expression profiles into a single ‘cluster’. In essence, the number of rows in the gene abundance matrix (*i.e.* the number of genes in Figure 1E) is reduced to two (or more) new variables that preserve the variances of gene expression, using dimensionality reduction techniques. As most single-cell experiments deal with tens of thousands of cells, linear methods such as principal component analysis (PCA) are inadequate, because they focus on placing the most distant cells apart and tend to place the remaining cells as one large cluster between them, making sub-clustering difficult. Therefore, non-linear methods exaggerating the similarity between cells are generally used, such as *t*-distributed Stochastic Neighbor Embedding (*t*SNE) and Uniform Manifold Approximation and Projection (UMAP). As a result, each cell is endowed with two (or more) new coordinates (Figure 1F), which can be used to visualize single cells as single points in the two-dimensional space of gene expression (Figure 1G). Although these methods make it easier to group cells into distinct clusters, the distances in such a non-linear space cannot be used as a proxy for gene expression differences.

Next, the neighborhood of the cells in this space is sub-divided using unsupervised clustering techniques (Figure 1G). A cluster of cells indicates the cells with highly similar gene expression profiles and may represent a cell ‘type’ (*e.g.* microglia or neuron) or ‘state’ (*e.g.* resting or activated). Each cluster is inspected for the enrichment or depletion of known ‘markers’ using gene annotation datasets (*e.g.* cluster 1 – microglia, cluster 2 – astrocytes, and cluster 3 – neurons) (Figure 1H).

Since each barcode can be traced back to the original tissue (Figures 1C and E), scRNA-seq or snRNA-seq data can be cross-examined with the sample labels, such as the age, genotype, and disease severity of the patient. Typical approaches include the comparison of cell type proportions, the detection of differentially expressed genes (DEGs) in each cell type, and pathway enrichment analyses such as gene set enrichment analysis (GSEA) (Figure 1I). With the above experimental and analytical techniques in mind, we now discuss the recent papers on neuro-immune interactions in fetal, young, and aged brains.

## Maternal immune activation impairs fetal brain development through cytokine signaling

Infection during pregnancy has long been associated with an increased risk of the unborn child's developing neuropsychiatric disorders in adulthood (Kwon et al. 2022). For example, epidemiological studies on the Spanish flu pandemic in the early twentieth century revealed a strong link between maternal immune activation and children's development of schizophrenia (Kepinska et al. 2020). Several recent studies also suggested that maternal infection may be a risk factor for autism spectrum disorder (ASD) in children (Patterson 2009; Lee et al. 2015). However, the molecular and cellular basis of these findings remained elusive.

Injecting poly(I:C) (synthetic double-stranded RNA, mimicking viral infection) intraperitoneally into pregnant mice (Smith et al. 2007) produces behavioral phenotypes in the offspring, which are similar to the symptoms of human ASD patients (Malkova et al. 2012). In this model, T helper 17 (Th17) cells in mothers secrete interleukin 17a (IL-17a) that crosses the placenta, enters the fetal brain, and activates IL-17a receptors on excitatory neuronal progenitors (Choi GB et al. 2016). Abnormally high IL-17a receptor activation in these cells impairs their differentiation and radial migration. The anomaly in the formation of cortical layers is observed specifically in the upper layers of the somatosensory cortex (Shin Yim et al. 2017). A similar anatomical phenotype is found in the brain of human ASD patients as patches of cortical dysplasia (Stoner et al. 2014), suggesting that this mouse model represents human ASD.

To understand the molecular and cellular mechanisms underlying this pathogenesis, Kalish and colleagues performed scRNA-seq of the fetal brains (embryonic days 14.5 and 18.5) developing in pregnant mice injected with poly(I:C) on embryonic day 12.5 (Kalish et al. 2021) (Table 1). They detected DEGs in the maternally inflamed fetal brain in all major cell types. Intriguingly, the difference in gene expression was evident only in males, in line with a male-higher prevalence of ASD in humans. In neurons, the genes required for protein synthesis were highly down-regulated. Further experiments revealed that the integrated stress response (ISR) pathway was responsible. PKR-like endoplasmic reticulum kinase (PERK) is activated in neuronal progenitors, which phosphorylates eukaryotic initiation factor 2α (eIF2α). Hyper-phosphorylated eIF2α inhibits the guanine nucleotide exchange factor (GEF) function of eIF2B, preventing the formation of the eIF2-GTP/Met-tRNAi ternary complex required for translational initiation. In short, viral infection raises IL-17a secretion in mothers, which crosses the placenta and fetal brain to activate the IL-17a receptors expressed in upper cortical neuronal progenitors, turning on the PERK-dependent ISR pathway, inhibiting protein synthesis and neuronal migration.

**Table 1.** Summary of key papers on single-cell and single-nucleus analysis of neuro-immune interactions in different pathological conditions. The methods are color-coded as in Figure 1. The acronyms used in this table are explained in the main text.

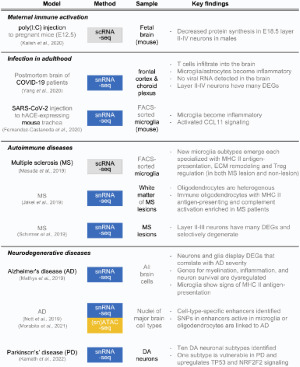

It is puzzling why the structural phenotype is manifest only in the sensory cortex because the expression of IL-17a receptor genes is not restricted to this region of the brain (Choi GB et al. 2016). Spatially-resolved transcriptomic analysis using the advanced techniques of spatial transcriptomics (Burgess 2019) will provide new data that may clarify the relationship between the region-specific structural deficits and the changes in gene expression.

## Peripheral inflammation in COVID-19 spreads to the brain

COVID-19 (coronavirus disease 2019), a respiratory disease, is caused by infection of severe acute respiratory syndrome coronavirus 2 (SARS-CoV-2) in humans. Severe COVID-19 symptoms stem from the hyperactive inflammatory response in the lower raspatory tract, leading to auto-immune attacks on the lungs. Comorbidity of COVID-19 includes cognitive impairment, which is colloquially referred to as ‘brain fog’ (Lempriere 2021; Solomon 2021; Ceban et al. 2022). Recent single-cell studies on human and mouse brains have provided the molecular basis of this neurological symptom.

Yang and colleagues performed snRNA-seq from the cerebral cortex and choroid plexus, which is the source of cerebrospinal fluid (CSF) at the blood–brain interface. The authors used the samples taken from patients who died from severe COVID-19 (Yang et al. 2021) (Table 1). Although there was no evidence of viral entry to the brain parenchyma, they found clear signs of neuroinflammation in the brain. Homeostatic gene expression was dysregulated, and the inflammatory genes were up-regulated in all cell types. In addition, peripheral T cells infiltrated the brain, indicating that the inflammation originating from the periphery spread to the brain. They identified the CCL and CXCL families of chemokines (chemotactic cytokines) as the key effector of this spread. Intriguingly, the excitatory neurons in the cortical layers 2-4, which are linked to cognitive function (Velmeshev et al. 2019), showed the most prominent alteration in gene expression, providing a potential molecular basis of the ‘brain fog’ in COVID-19. The study by Fullard and colleagues led to similar conclusions (Fullard et al. 2021).

Since most COVID-19 patients survive, it is unclear whether the above findings using post-mortem brains can be generalized. One recent study utilizing a mouse model of COVID-19 helped understand the neuroinflammatory mechanism in patients with mild COVID-19 (Fernandez-Castaneda et al. 2022). Fernández-Castañeda and colleagues used the adeno-associated virus (AAV)-mediated gene delivery method to express human ACE2 (hACE2), the human SARS-CoV-2 receptor, in the trachea and lungs of mice, allowing the viral infection (Table 1). As in humans, intratracheally injected SARS-CoV-2 remained in the lungs and did not enter the brain (Velmeshev et al. 2019; Fullard et al. 2021). Although these mice did not display apparent respiratory and neurological symptoms comparable to human patients, they did show clear molecular and cellular phenotypes. For example, the virus injection elevated cytokine concentrations in serum and CSF and induced microglia activation in the brain. The authors performed scRNA-seq of microglia isolated from the cerebral cortex and the corpus callosum (the major white matter of the brain) using fluorescence-activated cell sorting (FACS) and found the molecular signatures of dysregulated homeostatic gene expression and upregulated chemokine and inflammatory signaling. Further experiments identified the chemokine CCL11 as the key effector, as injecting CCL11 without the virus recapitulated the phenotypes, including microglia activation, oligodendrocyte death, and reduced hippocampal neurogenesis. Indeed, CCL11 is elevated in human patients with mild COVID-19, suggesting that the mechanism found in mice might also apply to humans.

In line with the above studies, a large-scale structural brain magnetic resonance imaging (MRI) study showed evidence for brain atrophy in human COVID-19 patients, in regions such as the orbitofrontal cortex, the primary olfactory cortex, and parahippocampal gyrus. Therefore, a strong immune activation in the periphery may lead to structural and functional deficits in the brain (Douaud et al. 2022).

## Microglia subtypes are clonally expanded in multiple sclerosis

Multiple sclerosis (MS) is an autoimmune disease of the CNS, in which auto-reactive myeloid cells attack myelin sheets, leading to motor disability and even death (Attfield et al. 2022). A recent scRNA-seq study in mice revealed that extensively heterogeneous myeloid cell subtypes exist in a healthy brain, suggesting a simple mechanism underlying the emergence of ‘disease-specific’ microglia (Jordao et al. 2019). This heterogeneity is not only found in the parenchymal macrophages (*i.e.* microglia), but also in non-parenchymal macrophages in the subarachnoid space, cerebral capillary vessels, and choroid plexus (which are collectively known as CNS-associated macrophages, or CAMs). During inflammation, some of these subtypes selectively multiply by clonal expansion.

Extensive scRNA-seq and snRNA-seq studies have been performed on MS patients, and several molecular and cellular characteristics were found (Jakel et al. 2019; Jordao et al. 2019; Masuda et al. 2019; Schirmer et al. 2019; Schafflick et al. 2020; Absinta et al. 2021; Kaufmann et al. 2021; Miedema et al. 2022). Masuda and colleagues performed scRNA-seq of FACS-sorted microglia from post-mortem human brains and found that several microglia subtypes are enriched in MS patients (Masuda et al. 2019) (Table 1). MS-specific microglia subtypes displayed the gene expression patterns indicative of major histocompatibility complex (MHC) class II-mediated antigen-presentation, extracellular matrix (ECM) remodeling, and regulatory T cell (Treg) modulation. This result suggests that microglia may present self-antigen and help peripheral T cells infiltrate the brain. A similar finding was made in experimental autoimmune encephalomyelitis (EAE) (Jordao et al. 2019), which is a mouse model of MS (Karpus 2020). In this model, however, it was the antigen-presenting machinery in the periphery that was required for the peripheral T cells to gain entry to the brain. Microglia's antigen presentation was not required for the development of EAE and only played a role in disease progression after T cell entry (Jordao et al. 2019) (Table 1).

To analyze T cells infiltrating the MS brain, Kaufmann and colleagues used scRNA-seq to investigate the molecular characteristics of CNS-colonizing T cells (Kaufmann et al. 2021), and Schafflick and colleagues performed scRNA-seq of leukocytes in the blood and CSF (Schafflick et al. 2020), both providing rich resources that can be used to develop therapeutic strategies to interfere with T cell infiltration (Kaufmann et al. 2021). To analyze neural cells, Absinta and colleagues performed snRNA-seq (Absinta et al. 2021) and found that T cell infiltration turns on the inflammatory genes in microglia and astrocytes. They pinpointed the complement C1q secreted from ‘microglia inflamed in MS (MIMS),’ an inflammatory microglia subtype, as the key mediator of MS progression. In accordance with this idea, the microglia-specific ablation of the C1q gene or the treatment with C1q neutralizing antibodies ameliorated the symptoms of EAE in mice. Microglia activation may precede the symptoms, as the inflammatory subtypes of microglia and CAMs were already enriched in the normal-appearing white matter of MS patients (Miedema et al. 2022).

Since oligodendrocytes are the primary target of autoimmune attack in MS, Jäkel and colleagues performed snRNA-seq from the white matter of post-mortem human brains to look at the oligodendrocytes in the MS brain (Jakel et al. 2019). The authors found that MS-enriched oligodendrocyte subtypes displayed the molecular characteristics of MHC class II-mediated antigen-presentation and complement activation, suggesting that oligodendrocytes may not only be a passive target of the autoimmune attack but also be an active participant in the pathogenesis (Table 1).

Finally, Schirmer and colleagues performed snRNA-seq of both gray and white matters of MS lesions to examine the disease-associated changes in all brain cell types, including neurons (Schirmer et al. 2019). An important finding was that the upper cortical neurons were the most affected neuronal subtype in MS, which showed the most numerous DEGs and clear signs of degeneration (Table 1). Other studies showed that the upper layer neurons were also selectively affected in maternal immune activation (Kalish et al. 2021) and COVID-19 (Yang et al. 2021), suggesting that they may be the most vulnerable neuronal subtype in neuroinflammation. Colonna and Brioschi speculated that the proximity of these neurons to the leptomeninges (pia and arachnoid), which are enriched with CAMs (Jordao et al. 2019) and blood vessels, is an intrinsic anatomical risk of these neurons, which increases the chance of encountering CAMs and peripheral leukocytes (Colonna and Brioschi 2020).

## Progression of neurodegenerative diseases is associated with neuroinflammation

Combinations of genetic and environmental risk factors influence the pathogenesis of diverse neurodegenerative diseases, such as Alzheimer's disease (AD), Parkinson's disease (PD), and amyotrophic lateral sclerosis (ALS). Although they have not been traditionally viewed as neuroinflammatory diseases, it is becoming increasingly clear that they have strong immunological components (Guzman-Martinez et al. 2019).

### Alzheimer's disease (AD)

Significant efforts have been made to understand the pathogenesis of AD at single-cell resolution (Del-Aguila et al. 2019; Grubman et al. 2019; Mathys et al. 2019; Lau et al. 2020; Zhou et al. 2020; Leng et al. 2021; Wang et al. 2022; Yang et al. 2022). For example, Mathys and colleagues performed snRNA-seq in post-mortem brains of AD patients with varying severity and profiled the gene expression status of all cell types (Mathys et al. 2019), following up on their original scRNA-seq study focused on microglia (Mathys et al. 2017). They found that neurons, microglia, oligodendrocytes, and astrocytes turn off homeostatic transcriptional programs and activate inflammatory gene expression, clarifying the link between neuroinflammation and AD. For example, one AD-enriched microglia subtype expressed the genes associated with MHC class II-mediated antigen-presentation in a disease stage-dependent manner. Neurons were the most affected cell type, although the layer identities were not analyzed in this study. An intriguing finding was that the AD-associated cell subtypes showed sex differences, enriched in females (Table 1).

Recently, Nott and colleagues used fluorescence-activated nuclei sorting (FANS) to isolate the nuclei of neurons (NEUN^+^), astrocytes (NEUN^neg^ LHX2^+^), oligodendrocytes (OLIG2^+^), and microglia (PU.1^+^) to collect the nuclei of these cell types and performed ATAC-seq to produce a genome-wide survey of cell type-specifically open chromatin regions in AD brain cells (Nott et al. 2019). Combining this with the databases of AD-associated single nucleotide polymorphisms (SNPs), the authors generated a deeper and multidimensional dataset, from which *cis*-regulatory elements activated in each cell type in healthy and AD brains could be analyzed. This study led to an intriguing model, in which SNPs in enhancers that are active in microglia but not in neurons are linked with an increased risk of AD, adding to the accumulating evidence for the immunological contribution to AD pathogenesis. More recently, Morabito and colleagues performed single–nucleus ATAC–seq (snATAC–seq) and snRNA–seq from the same AD brains to extend this finding to single–cell resolution and identified many cell type–specific and AD–associated *cis–*regulatory elements (Morabito et al. 2021) (Table 1).

### Parkinson's disease (PD)

PD is a disease characterized by a progressive loss of dopaminergic (DA) neurons in the substantia nigra pars compacta (SNpc) of the midbrain. Smajić performed snRNA-seq of post-mortem brains from patients with idiopathic PD and identified PD-specific neuronal and glial subtypes. The authors provided evidence for ‘pan-glial’ immune activation, revealing a clear link between PD and neuroinflammation (Smajic et al. 2022). In a more recent, larger-scale study, Kamath and colleagues performed snRNA-seq using over 387,000 nuclei containing those of over 22,000 DA neurons (Kamath et al. 2022). From this dataset, the authors identified a DA neuronal subtype that is specifically lost in PD, perhaps due to ‘pan-glial’ immune activation in PD brains. They identified ten DA neuronal subtypes in the normal brain and found that only one type was susceptible to degeneration in PD. Having found the molecular features of this DA neuronal subtype, which include upregulated TP53 and NRF2F, the authors identified their location in the ventral region of the SNpc, revealing the molecular and anatomical bases of PD initiation and progression.

## Discussion

Together, single-cell and single-nucleus studies have revealed a strong association between neuroinflammation and different modalities of CNS diseases and provided rich open resources inviting new hypotheses for pathogenesis and therapeutic strategies. The raw sequencing data and the gene-level expression matrices are usually accessible in public data repositories, such as gene expression omnibus (GEO) (https://www.ncbi.nlm.nih.gov/geo/), and in many cases, the cell type assignment information is also available. Typically, users can download the gene expression matrix and cell type information and use software packages, such as *Seurat* (Hao et al. 2021) in R or *scanpy* (Wolf et al. 2018) in Python, to perform customized analyses. Convenient interactive tools that visualize single cells and nuclei in the two-dimensional gene expression space are also available, for example, the web-based UCSC Cell Browser (https://www.cells.ucsc.edu) and installed Loupe Browser (10x Genomics). The former is particularly useful, as it curates the data from the latest papers and provides an interactive viewer in the exact format published in the original articles. Additionally, the gene expression (including dimension-reduced) and cell information matrices are stored in a systematic format, making downstream analyses by end-users much easier.

Common themes emerge from the studies of diseases directly caused by neuroinflammation, such as maternal immune activation, COVID-19, and MS. Peripherally originating inflammation spreads to the CNS, resulting in a global change in gene expression status in all cell types. Neurons, macroglia (astrocytes and oligodendrocytes), microglia, and CAMs down-regulate their respective homeostatic gene expression and up-regulate genes associated with inflammation. Microglia, which appear to be the main driver of disease progression, are composed of molecularly distinct subtypes, and upon encountering inflammatory insults the subtypes specialized for antigen-presentation and secreting inflammatory cytokines are clonally expanded. Astrocytes precipitate neuroinflammation by receiving and secreting inflammatory cytokines, eventually leading to the degeneration of oligodendrocytes and neurons and the deterioration of brain function. Symptoms may display sex differences, as in the case of maternal immune activation and multiple sclerosis. In both developing and mature brains, the upper layer cortical neurons, which may be more accessible to peripheral cytokines and leukocytes, appear to be more vulnerable, which could contribute to cognitive impairment associated with infection. How and which peripheral inflammatory signals and/or cells overcome the blood-placental and blood–brain barrier and reach the brain parenchyma is an important area for future studies.

Neuroinflammation also regulates the pathogenesis of neurodegenerative diseases, but in a different way. In this case, neuroinflammation is triggered by disease-specific neurogenerative burdens, such as the accumulation of intracellular aggregates (*e.g.* hyper-phosphorylated Tau in AD, RNA granules in ALS, and Lewy body in PD) or extracellular plaques, such as amyloid plaques. This may cause CNS-originating inflammatory activation.

Since neurons in the brain express a battery of cytokines and cytokine receptors even without inflammatory insults, it is an intriguing possibility that cytokine signaling plays regulatory roles in normal brain function. In line with this idea, evidence implicated cytokines in anxiety-like behavior (Alves de Lima et al. 2020) and the formation of contextual fear memory (Herz et al. 2021) in mice. Therefore, various immunological challenges may interfere with these normal cytokine functions in the brain, with or without activating the innate immune response. We are continuously exposed to pathogens, yet they do not generally cause strong neuroinflammation. Therefore, it is critical to understand how inflammatory insults are processed under normal and pathological circumstances.

The single-cell studies of fetal mouse and human brains showed that the neural progenitors in the developing embryo express cytokine receptors, suggesting an unexplored possibility that ‘inflammatory’ cytokines regulate normal brain development, just as shown for chemokines two decades ago (Tran and Miller 2003). Indeed, maternal immune activation increases cytokine concentration in the fetal brain and impairs the migration of the receptor-bearing neuronal progenitors (Choi GB et al. 2016). Astrocytes in the developing brain also secrete cytokines, which act on cytokine receptors on microglia that participate in synapse elimination and neural circuit development (Vainchtein et al. 2018). In this sense, it is noteworthy that the maternal microbiome modulates fetal brain development in mice (Vuong et al. 2020). Identifying cytokines that regulate normal brain development will be an exciting subject that can be studied using single-cell approaches.
